# The Effect of Bevacizumab Therapy on Reducing Erythrocyte Transfusion Requirements and Preventing Iron Deficiency in Patients With Hereditary Hemorrhagic Telangiectasia

**DOI:** 10.7759/cureus.102365

**Published:** 2026-01-27

**Authors:** Öznur Aydın, Fatoş Dilan Köseoğlu, Selim Sayın, Hilmi Erdem Gözden, Onur Şahin, Ünal Ataş, Derya Deniz Kürekci, Sude Hatun Aktimur, Meltem Aylı, Mehmet Turgut, Serkan Güven

**Affiliations:** 1 Department of Hematology, Faculty of Medicine, Samsun University, Samsun, TUR; 2 Department of Hematology, Faculty of Medicine, İzmir Bakırçay University, Çiğli Training and Research Hospital, İzmir, TUR; 3 Department of Hematology, Gülhane Training and Research Hospital, Ankara, TUR; 4 Department of Hematology, Bursa Yüksek İhtisas Education and Research Hospital, Bursa, TUR; 5 Department of Hematology, Faculty of Medicine, Ondokuz Mayıs University, Samsun, TUR; 6 Department of Hematology, Faculty of Medicine, Akdeniz University, Antalya, TUR; 7 Department of Hematology, Samsun City Hospital, Samsun, TUR; 8 Department of Hematology, Çanakkale Mehmet Akif Ersoy State Hospital, Çanakkale, TUR

**Keywords:** anemia, bevacizumab, epistaxis, hereditary hemorrhagic telangiectasia, iron deficiency, transfusion, vegf

## Abstract

Objective: This multicenter retrospective cohort study aimed to evaluate the association between intravenous (IV) bevacizumab therapy and iron-related parameters and transfusion burden in patients with hereditary hemorrhagic telangiectasia (HHT) in Turkey.

Methods: Thirty-five genetically or clinically confirmed HHT patients from tertiary centers were included. Patients receiving IV bevacizumab (n=15) were compared with those without bevacizumab exposure (n=20). Longitudinal hematologic parameters, annual IV iron dose, erythrocyte suspension (ES) transfusion volume, and bleeding frequency were recorded. In the bevacizumab group, pre- and post-treatment values were compared.

Results: Before treatment, patients who subsequently received bevacizumab had higher ES transfusion requirements than untreated patients (median 6 vs. 2 units; p=0.029). After bevacizumab therapy, IV iron requirement decreased (median 5000 mg to 2000 mg; p=0.005) and ES transfusion burden declined (median 6 to 0 units; p=0.002). Compared with the non-bevacizumab group, treated patients had higher final hemoglobin (p=0.042) and ferritin levels (p=0.048), while transferrin saturation was higher in the untreated group (p=0.047).

Conclusion: In this multicenter retrospective cohort, IV bevacizumab therapy was associated with improved iron parameters and reduced transfusion requirements in patients with HHT-related bleeding.

## Introduction

Hereditary hemorrhagic telangiectasia (HHT) is an autosomal dominant vascular disorder that, although rare, can exert significant effects on both quality of life and life expectancy [1,2]. The clinical presentation is primarily characterized by recurrent epistaxis, gastrointestinal bleeding, and iron deficiency anemia [3]. According to epidemiological estimates, the prevalence of HHT is approximately 1 in 5,000-8,000 individuals [4]. The clinical spectrum ranges from asymptomatic individuals to patients with life-threatening complications, highlighting the need for a multidisciplinary management approach [5].

The genes most commonly involved in HHT pathogenesis are ENG and ACVRL1, with less frequent variants including SMAD4 and GDF2/BMP9 [6]. Mutations in these genes disrupt transforming growth factor-β (TGF-β) signaling pathways, leading to the development of arteriovenous malformations (AVMs) in visceral organs such as the lungs, liver, and brain, as well as mucocutaneous telangiectasias [7,8].

The diagnosis of HHT is primarily based on the clinical Curaçao criteria, which comprise four main features: spontaneous and recurrent epistaxis; mucocutaneous telangiectasias at characteristic sites such as the lips, oral cavity, fingers, and nose; the presence of visceral AVMs involving organs such as the lungs, liver, brain, or gastrointestinal tract; and a first-degree relative with HHT [9]. The presence of at least three criteria establishes a definite diagnosis, two criteria indicate a possible diagnosis, and fewer than two criteria make HHT unlikely. These clinically based criteria are widely used in practice and may be supported by genetic testing, particularly in borderline cases or during family screening [10].

One of the cornerstone components of HHT management is the control of chronic and recurrent bleeding. Treatment options include nasal laser photocoagulation, bipolar cauterization, topical agents, antifibrinolytics such as tranexamic acid, endovascular embolization, and systemic therapies when indicated [11]. International guidelines provide standardized recommendations regarding screening strategies and imaging approaches for high-risk organs, epistaxis management, follow-up of visceral AVMs, and pregnancy-specific monitoring [12,13]. However, these modalities are often insufficient in patients with refractory or frequent epistaxis, leading to chronic iron deficiency and recurrent transfusion requirements. These clinical challenges have increased interest in targeted anti-angiogenic therapies [14].

Bevacizumab is a therapeutic agent that exerts its effects through inhibition of vascular endothelial growth factor (VEGF), thereby stabilizing vascular structures and controlling bleeding. Due to its efficacy in reducing bleeding risk, bevacizumab has emerged as a promising treatment option for patients with HHT [15]. Available studies have demonstrated that intravenous (IV) bevacizumab reduces epistaxis severity, decreases the need for IV iron supplementation, and significantly lowers erythrocyte transfusion requirements [16,17]. In contrast, studies investigating locally administered bevacizumab (nasal/intranasal/submucosal) have yielded heterogeneous results. While small case series have reported marked clinical improvement, randomized controlled trials have shown limited or statistically non-significant effects on epistaxis frequency and severity [18-20]. These inconsistencies suggest that treatment efficacy may depend on the route of administration, dosing intervals, and patient selection.

In recent years, the use of additional immunomodulatory or anti-angiogenic agents in the treatment of HHT has also been explored. Thalidomide and pomalidomide may provide benefit in bleeding control in selected refractory cases; however, their long-term toxicity profiles necessitate careful evaluation [21,22]. Targeted approaches such as tacrolimus and sirolimus, supported by more limited evidence, remain areas of ongoing investigation [23,24]. In this context, bevacizumab continues to represent a strong therapeutic option that can be used alone or in combination with supportive treatments in appropriately selected patients.

Therefore, the aim of this study was to evaluate the association between systemic IV bevacizumab therapy and IV iron replacement requirements and erythrocyte transfusion burden in patients with HHT in a multicenter, real-world setting in Turkey. By addressing the lack of national data and providing real-world evidence consistent with the international literature, this study seeks to contribute to a better understanding of the role of systemic bevacizumab in the management of bleeding-related complications in HHT.

## Materials and methods

This study was designed as a multicenter, retrospective cohort investigation involving patients diagnosed with HHT who were followed at various tertiary healthcare centers across Turkey. Patients were included if they had a definite diagnosis of HHT based on the Curaçao diagnostic criteria (≥3 criteria), as previously described in the study by Shovlin et al. [9], or if the diagnosis was confirmed by molecular diagnostic testing. Patients with insufficient clinical or laboratory data that precluded longitudinal evaluation were excluded. A total of 35 patients were evaluated, of whom 15 had received IV bevacizumab therapy, while 20 had no history of bevacizumab treatment and were considered the comparison group.

Patients were selected for systemic bevacizumab therapy based on recurrent or refractory bleeding requiring frequent IV iron replacement and/or erythrocyte transfusions, as determined by the treating physician during routine clinical follow-up.

To ensure consistency and data harmonization across participating centers, demographic and clinical variables were collected using a standardized data collection form. Recorded variables included age, sex, age at diagnosis, family history of HHT, pathogenic mutations detected in HHT-related genes (e.g., ENG, ACVRL1, SMAD4), presence of epistaxis, bleeding frequency as documented during routine clinical follow-up, and history of thrombotic events.

Standardized bleeding severity scores (such as the Epistaxis Severity Score) were not uniformly available across participating centers. Therefore, bleeding severity was indirectly assessed using surrogate markers, including IV iron requirements and erythrocyte transfusion burden.

Hematologic and iron-related parameters were evaluated based on measurements obtained during routine follow-up. Hemoglobin levels, ferritin levels, serum iron, transferrin saturation, annual IV iron therapy dose, and annual erythrocyte suspension (ES) transfusion volume were extracted from institutional electronic medical records and entered into the study database. Annual IV iron requirements and ES transfusion burden were calculated based on cumulative doses or units administered within a 12-month period.

Among patients receiving bevacizumab, treatment response was assessed by comparing hematologic and iron-related parameters between pre-treatment and post-treatment periods within the same individual. The duration of bevacizumab therapy was recorded for each patient. Follow-up duration varied according to individual clinical course and center-specific practice patterns.

Due to the retrospective and multicenter nature of the study, bevacizumab dosing regimens and treatment schedules were not standardized and varied across centers. In routine clinical practice, bevacizumab was administered intravenously at doses generally ranging from commonly used doses reported in the literature, with induction and maintenance schedules determined by the treating physician based on bleeding severity and clinical response.

The primary outcome measures were defined as changes in annual IV iron replacement requirements and annual ES transfusion needs between the pre- and post-treatment periods. Secondary outcome measures included changes in hematologic and iron parameters, such as hemoglobin level, ferritin level, and transferrin saturation, as well as changes in bleeding frequency. In addition, the most recent hematologic parameters were descriptively compared between patients who received bevacizumab and those who did not, in order to evaluate treatment effects under real-world clinical conditions.

Data collection was performed independently at each participating center, and data accuracy was ensured through centralized review and verification by the coordinating research team. All laboratory measurements were conducted in accredited institutional laboratories using routine clinical methods and locally accepted reference ranges.

Ethical approval for the study was obtained from the Samsun University Non-Interventional Clinical Research Ethics Committee (Approval No: 2025/6/14; Date: August 6, 2025). The study was conducted in accordance with the principles of the Declaration of Helsinki and institutional standards applicable to retrospective research.

Statistical analysis

Statistical analyses were performed using IBM SPSS Statistics for Windows, Version 25 (Released 2017; IBM Corp., Armonk, New York, United States). Categorical variables were presented as numbers (n) and percentages (%), while continuous variables were summarized using descriptive statistics, including mean ± standard deviation (SD) and median (minimum-maximum), depending on distributional characteristics.

For comparisons between groups, the Mann-Whitney U test was applied when parametric assumptions were not met. Fisher’s exact chi-square test was used for the analysis of categorical variables when appropriate. All analyses were conducted using two-tailed tests, and a p-value of <0.05 was considered statistically significant.

## Results

Table 1 summarizes the sociodemographic and clinical characteristics of the 35 patients included in the study. Twenty patients had no history of bevacizumab therapy, while 15 patients had previously received bevacizumab. Patients in the bevacizumab group were significantly older and had a higher age at diagnosis compared with those without bevacizumab exposure (p = 0.043 and p = 0.002, respectively). The distribution of family history of HHT, presence of epistaxis. and mutation subtype (ENG vs. ACVRL1) did not differ significantly between the two groups (p > 0.05). However, thrombosis was more frequent in patients with a history of bevacizumab use (20%). This difference did not reach statistical significance (p = 0.070).

**Table 1:** Comparison of Sociodemographic and Clinical Characteristics According to Bevacizumab History Groups a: Mann–Whitney U test; b: Fisher’s exact test; p < 0.05 was considered statistically significant.

		Bevacizumab History			
Variables	Total N=35	No N=20	Yes N=15	p	U/X^2^
Age					
Mean±SD	55.17±14.08	51.20±14.79	60.46±11.49	0.043^a^	-2.020
Median (min-max)	57.0 (24-76)	50.5 (24-73)	61.0 (39-76)		
Age at diagnosis					
Mean±SD	47.62±13.31	41.75±11.25	55.46±11.98	0.002^a^	-2.952
Median (min-max)	45.0 (18-74)	43.0 (18-67)	55.0 (38-74)		
Family history of HHT					
No	1 (2.9)	1 (5.0)	0 (0.0)	1.000^b^	0.721
Yes	33 (97.1)	19 (95.0)	14 (100.0)		
Genetic mutation					
ENG	3 (30.0)	2 (40.0)	1 (20.0)	0.500^b^	0.476
ACVRL1	7 (70.0)	3 (60.0)	4 (80.0)		
Epistaxis					
No	3 (8.6)	3 (15.0)	0 (0.0)	0.244^b^	2.461
Yes	32 (91.4)	17 (85.0)	15 (100.0)		
History of thrombosis					
No	32 (91.4)	20 (100.0)	12 (80.0)	0.070^b^	4.375
Yes	3 (8.6)	0 (0.0)	3 (20.0)		

Table 2 presents the comparison of iron requirements, transfusion burden, and bleeding frequency before bevacizumab therapy. The annual IV iron dose tended to be higher among patients who eventually received bevacizumab, although the difference was not statistically significant (p = 0.100). However, ES transfusion requirements were significantly greater in the bevacizumab group prior to treatment initiation (median 6 vs. 2 units; p = 0.029). Bleeding frequency showed no significant difference between groups (p = 0.892).

**Table 2:** Comparison of Pre-bevacizumab Iron Requirement, Bleeding Frequency, and Transfusion Need Between Patient Groups a: Mann–Whitney U test; p < 0.05 was considered statistically significant.

		Bevacizumab History			
Variables	Total N=35	No N=20	Yes N=15	p	U
Annual intravenous iron dose (mg)					
Median (min-max)	4000.0 (0-48000)	2500.0 (0-15000)	5000.0 (0-48000)	0.100^a^	-1.646
Erythrocyte suspension transfusion amount					
Median (min-max)	4.0 (0-32)	2.0 (0-32)	6.0 (0-24)	0.029^a^	-2.187
Bleeding frequency					
Median (min-max)	3.5 (0.0-48.0)	3.0 (2.0-8.0)	4.0 (0.0-48.0)	0.892^a^	-0.135

Table 3 shows the pre- and post-treatment comparison within the bevacizumab-treated group. A significant decline in both annual IV iron requirement (median 5000 mg to 2000 mg; p = 0.005) and ES transfusion need (median 6 to 0 units; p = 0.002) was observed following bevacizumab therapy. These results indicate that bevacizumab substantially reduced iron deficiency-related treatment burden. In the bevacizumab-treated group, the duration of therapy ranged between 6 and 24 months.

**Table 3:** Comparison of Parameters Before and After Bevacizumab Therapy in the Bevacizumab-Treated Group Wilcoxon test; p < 0.05 was considered statistically significant.

Variables	Before Bevacizumab Therapy (N=15)	After Bevacizumab Therapy (N=15)	p	U
Intravenous iron therapy dose (mg) Median (min–max)	5000.0 (0–48000)	2000.0 (0–12000)	0.005	-2.825
Erythrocyte suspension transfusion amount Median (min–max)	6.0 (0–24)	0.0 (0-5)	0.002	-2.156

Table 4 compares the most recent hematologic and iron parameters according to bevacizumab history. Significant differences were found in transferrin saturation (p = 0.047), ferritin levels (p = 0.048), and hemoglobin levels (p = 0.042). Patients treated with bevacizumab demonstrated higher ferritin and hemoglobin levels, whereas transferrin saturation was higher among those who did not receive bevacizumab. Overall, these findings suggest that bevacizumab therapy is associated with improved iron storage and hemoglobin recovery.

**Table 4:** Comparison of Latest Hematologic and Iron Parameters According to Bevacizumab History a: Mann–Whitney U test; p < 0.05 was considered statistically significant.

		Bevacizumab History			
Variables	Total N=35	No N=20	Yes N=15	p	U
Latest transferrin saturation					
Median (min-max)	15.0 (3.6-35.0)	19.9 (3.6-35.0)	15.0 (4.2-34.0)	0.047^a^	-1.334
Latest ferritin level					
Median (min-max)	26.0 (4.0-323.0)	20.8 (4.0-323.0)	27.0 (4.0-204.0)	0.048^a^	-1.330
Latest hemoglobin level					
Median (min-max)	12.1 (7.4-16.9)	10.4 (7.6-16.9)	13.6 (7.4-15.8)	0.042^a^	-1.850

## Discussion

In our study, bevacizumab therapy was shown to significantly reduce the requirements for IV iron replacement and ES transfusions in patients with HHT. In addition, post-treatment evaluations revealed that hemoglobin and ferritin levels were higher in patients receiving bevacizumab compared with those who did not, whereas transferrin saturation was higher in the untreated group. Notably, although the pre-treatment transfusion requirement was significantly higher in the bevacizumab-treated group, a marked reduction in replacement needs was observed after therapy. This finding suggests that bevacizumab may effectively reduce replacement requirements even in patients with a more severe disease phenotype and can be interpreted as providing a clinically meaningful improvement in the management of chronic bleeding and anemia associated with HHT. However, given the retrospective and observational nature of the study, these findings should be interpreted as associations rather than evidence of causality.

In HHT, chronic and recurrent bleeding is not limited to local clinical manifestations such as epistaxis and gastrointestinal bleeding but leads over time to iron deficiency anemia, recurrent erythrocyte transfusions, and increased healthcare utilization [25]. Therefore, treatment approaches in HHT that focus solely on symptom control are often insufficient, and systemic treatment strategies aimed at reducing disease burden and healthcare system impact have gained increasing importance. In this context, anti-angiogenic approaches targeting vascular pathophysiology are being used in clinical practice, particularly in selected patient groups whose bleeding cannot be adequately controlled despite standard local and supportive treatments, and are being evaluated in a growing number of studies [26,27].

In the InHIBIT-Bleed study, which includes the largest patient cohort reported to date on this topic, a total of 238 patients from 12 centers were evaluated. The study demonstrated that systemic bevacizumab therapy was highly effective in reducing both epistaxis and gastrointestinal bleeding in patients with HHT. At the end of the study, the mean hemoglobin level increased by 3.2 g/dL, while the mean epistaxis severity score decreased by 3.4 points. In addition, requirements for erythrocyte transfusions and IV iron therapy were significantly reduced. These effects were observed independently of the underlying genetic mutation, and no significant difference in treatment response was detected between patients carrying ENG or ACVRL1 mutations. Patients receiving continuous maintenance therapy achieved more stable hemoglobin levels and lower epistaxis scores compared with those treated with intermittent protocols [16].

These findings are further supported by a meta-analysis published in 2023. This analysis, which included seven clinical studies, demonstrated that bevacizumab therapy significantly reduced the Epistaxis Severity Score in patients with HHT; however, no statistically significant effect was observed on the duration or frequency of epistaxis. Importantly, adverse event rates were not increased compared with the control group, suggesting that bevacizumab may represent a safe treatment option [28].

Similarly, the BEST study, which serves as a continuation of the randomized phase II BABH trial, evaluated the one-year efficacy of bevacizumab in patients with HHT who had severe epistaxis and gastrointestinal bleeding. In this study, the majority of patients receiving bevacizumab (approximately 86%) achieved a reduction of more than 50% in erythrocyte transfusion requirements and an average increase of 30 g/L in hemoglobin levels. These results indicate a rapid and pronounced clinical improvement, particularly in patients with severe gastrointestinal bleeding. In addition, patients who benefited from bevacizumab in the earlier BABH study were reported to continue to show improvement with additional induction and maintenance therapies. The study also emphasized the challenges of conducting randomized controlled trials in rare diseases, highlighting small sample sizes, heterogeneous patient populations, and ethical constraints as major limitations. Overall, the BEST study supports the conclusion that bevacizumab significantly reduces transfusion requirements in severe HHT-related bleeding and underscores the importance of real-world data in this patient population [17,29].

In an assessment published by the European Reference Network for Rare Vascular Diseases (VASCERN), it was emphasized that bevacizumab should be considered particularly in cases that are refractory to standard approaches and associated with persistent transfusion requirements. Given that none of the currently available mechanism-based therapies for HHT can induce regression of AVMs, treatment strategies in HHT are primarily guided by clinical manifestations. However, the frequent recurrence observed within approximately one year after bevacizumab therapy has raised the need for maintenance dosing. In the absence of randomized controlled trials and in light of limited long-term safety data, bevacizumab is regarded not as a curative therapy for HHT but as an effective supportive treatment option that facilitates bleeding control in selected patients [14].

In addition to its clinical burden, HHT poses a substantial psychosocial and healthcare system challenge. Patients frequently require repeated hospital visits for transfusion support, iron infusions, epistaxis control procedures, and imaging surveillance for visceral AVMs, which collectively result in significant time, cost, and quality-of-life implications for both patients and caregivers. From a health system perspective, recurrent bleeding and untreated iron deficiency contribute to increased emergency department visits, longer hospital stays, and greater healthcare resource utilization. Therefore, therapeutic strategies that not only reduce bleeding but also decrease the need for parenteral iron and ES transfusions may offer broad benefits that extend beyond hematologic improvement, directly impacting patient-centered outcomes and healthcare efficiency. In this context, a cost-effectiveness analysis published in 2024 demonstrated that systemic bevacizumab therapy is not only clinically effective but also a cost-saving intervention for healthcare systems in the long term. According to this study, the addition of bevacizumab to standard treatment resulted in an average annual reduction of 133 hours in healthcare utilization per patient. This finding remained consistent across scenario analyses using different iron preparations and did not alter the cost-effectiveness of the intervention. Moreover, sensitivity analyses showed that no single variable was sufficient to shift the model outcome in favor of standard treatment alone [30].

Therefore, bevacizumab should be considered an important systemic treatment option for moderate to severe HHT-related bleeding, not only because of its clinical efficacy but also in view of its potential to reduce healthcare utilization and its contributions to sustainability. Improving access to treatment and increasing awareness among healthcare professionals are of particular importance for improving clinical outcomes in this patient population.

This study has several noteworthy strengths. First, it represents one of the few multicenter cohorts from Turkey that systematically evaluates the use of systemic bevacizumab in HHT, thereby contributing geographically distinct real-world data to the existing literature. Second, the use of clearly defined and clinically relevant endpoints, such as annual IV iron requirements and ES transfusion burden, provides a transparent and easily interpretable measure of treatment effect in routine clinical practice. Third, the inclusion of a comparison group not receiving bevacizumab allowed the hematologic improvements observed in treated patients to be evaluated in relation to the natural disease course under standard care.

Nevertheless, several important limitations should be acknowledged. The retrospective design and relatively small sample size limit the statistical power to detect modest differences and preclude robust subgroup analyses according to genotype or specific AVM phenotypes. In addition, although reflective of real-world practice, variability in the duration of bevacizumab therapy across participating centers may have contributed to heterogeneity in treatment exposure. Finally, the absence of validated epistaxis severity scores and detailed quality-of-life assessments restricts a comprehensive evaluation of the symptomatic benefits experienced by patients. In addition, adverse events related to bevacizumab therapy could not be systematically evaluated due to the retrospective design and incomplete documentation across centers. Finally, as with all observational studies, causal inferences regarding treatment effects cannot be definitively established.

## Conclusions

From a clinical perspective, our findings underscore the importance of systematic and longitudinal documentation of iron parameters, transfusion requirements, and bleeding history in patients with HHT who are being considered for bevacizumab therapy, as this approach enables objective evaluation of treatment response and supports timely clinical decision-making. Future research should focus on multicenter, prospective cohort studies incorporating standardized dosing protocols, clearly defined criteria for treatment initiation and discontinuation, and patient-reported outcome measures. Although randomized controlled trials may be difficult to conduct in this rare and heterogeneous disease, harmonized real-world data registries and collaborative research networks may substantially strengthen the evidence base and inform long-term management strategies.

## References

[1] Zarrabeitia R, Fariñas-Álvarez C, Santibáñez M, Señaris B, Fontalba A, Botella LM, Parra JA (2017). Quality of life in patients with hereditary haemorrhagic telangiectasia (HHT). Health Qual Life Outcomes.

[2] Droege F, Thangavelu K, Stuck BA, Stang A, Lang S, Geisthoff U (2018). Life expectancy and comorbidities in patients with hereditary hemorrhagic telangiectasia. Vasc Med.

[3] Jackson SB, Villano NP, Benhammou JN, Lewis M, Pisegna JR, Padua D (2017). Gastrointestinal manifestations of hereditary hemorrhagic telangiectasia (HHT): a systematic review of the literature. Dig Dis Sci.

[4] Govani FS, Shovlin CL (2009). Hereditary haemorrhagic telangiectasia: a clinical and scientific review. Eur J Hum Genet.

[5] Alkhalid Y, Darji Z, Shenkar R, Clancy M, Dyamenahalli U, Awad IA (2023). Multidisciplinary coordinated care of hereditary hemorrhagic telangiectasia (Osler-Weber-Rendu disease). Vasc Med.

[6] McDonald J, Wooderchak-Donahue W, VanSant Webb C, Whitehead K, Stevenson DA, Bayrak-Toydemir P (2015). Hereditary hemorrhagic telangiectasia: genetics and molecular diagnostics in a new era. Front Genet.

[7] Shovlin CL (2010). Hereditary haemorrhagic telangiectasia: pathophysiology, diagnosis and treatment. Blood Rev.

[8] Danesino C, Cantarini C, Olivieri C (2023). Hereditary hemorrhagic telangiectasia in pediatric age: focus on genetics and diagnosis. Pediatr Rep.

[9] Shovlin CL, Guttmacher AE, Buscarini E (2000). Diagnostic criteria for hereditary hemorrhagic telangiectasia (Rendu-Osler-Weber syndrome). Am J Med Genet.

[10] Floria M, Năfureanu ED, Iov DE (2022). Hereditary hemorrhagic telangiectasia and arterio-venous malformations: from diagnosis to therapeutic challenges. J Clin Med.

[11] Kritharis A, Al-Samkari H, Kuter DJ (2018). Hereditary hemorrhagic telangiectasia: diagnosis and management from the hematologist's perspective. Haematologica.

[12] Faughnan ME, Mager JJ, Hetts SW (2020). Second international guidelines for the diagnosis and management of hereditary hemorrhagic telangiectasia. Ann Intern Med.

[13] Al-Samkari H (2021). Hereditary hemorrhagic telangiectasia: systemic therapies, guidelines, and an evolving standard of care. Blood.

[14] Dupuis-Girod S, Shovlin CL, Kjeldsen AD (2022). European Reference Network for Rare Vascular Diseases (VASCERN): when and how to use intravenous bevacizumab in Hereditary Haemorrhagic Telangiectasia (HHT)?. Eur J Med Genet.

[15] Robert F, Desroches-Castan A, Bailly S, Dupuis-Girod S, Feige JJ (2020). Future treatments for hereditary hemorrhagic telangiectasia. Orphanet J Rare Dis.

[16] Al-Samkari H, Kasthuri RS, Parambil JG (2021). An international, multicenter study of intravenous bevacizumab for bleeding in hereditary hemorrhagic telangiectasia: the InHIBIT-Bleed study. Haematologica.

[17] Dupuis-Girod S, Rivière S, Lavigne C (2023). Efficacy and safety of intravenous bevacizumab on severe bleeding associated with hemorrhagic hereditary telangiectasia: a national, randomized multicenter trial. J Intern Med.

[18] García-Martín E, Pernía-López S, Martínez-Ortega PA, Monje B, Ruiz-Martínez C, Sanjurjo-Saez M (2019). Intranasal bevacizumab treatment on epistaxis in hereditary haemorrhagic telangiectasia: a case report. Eur J Hosp Pharm.

[19] Riss D, Burian M, Wolf A, Kranebitter V, Kaider A, Arnoldner C (2015). Intranasal submucosal bevacizumab for epistaxis in hereditary hemorrhagic telangiectasia: a double-blind, randomized, placebo-controlled trial. Head Neck.

[20] Whitehead KJ, Sautter NB, McWilliams JP (2016). Effect of topical intranasal therapy on epistaxis frequency in patients with hereditary hemorrhagic telangiectasia: a randomized clinical trial. JAMA.

[21] Invernizzi R, Quaglia F, Klersy C (2015). Efficacy and safety of thalidomide for the treatment of severe recurrent epistaxis in hereditary haemorrhagic telangiectasia: results of a non-randomised, single-centre, phase 2 study. Lancet Haematol.

[22] Al-Samkari H, Kasthuri RS, Iyer VN (2024). Pomalidomide for epistaxis in hereditary hemorrhagic telangiectasia. N Engl J Med.

[23] Hessels J, Kroon S, Boerman S (2022). Efficacy and safety of tacrolimus as treatment for bleeding caused by hereditary hemorrhagic telangiectasia: an open-label pilot study. J Clin Med.

[24] Ruiz S, Zhao H, Chandakkar P (2020). Correcting Smad1/5/8, mTOR, and VEGFR2 treats pathology in hereditary hemorrhagic telangiectasia models. J Clin Invest.

[25] Al-Samkari H, Mayne TJ, Troutt M, Patle H, Clancy M, Duhaime E (2025). Characterizing the healthcare utilization and costs of hereditary hemorrhagic telangiectasia. Am J Hematol.

[26] Al-Samkari H, Kritharis A, Rodriguez-Lopez JM, Kuter DJ (2019). Systemic bevacizumab for the treatment of chronic bleeding in hereditary haemorrhagic telangiectasia. J Intern Med.

[27] Iyer VN, Apala DR, Pannu BS (2018). Intravenous bevacizumab for refractory hereditary hemorrhagic telangiectasia-related epistaxis and gastrointestinal bleeding. Mayo Clin Proc.

[28] Chen H, Zhang Z, Chen X (2023). Meta-analysis of efficacy and safety of bevacizumab in the treatment of hereditary hemorrhagic telangiectasia epistaxis. Front Pharmacol.

[29] Dupuis-Girod S, Decullier E, Rivière S (2025). BEST study: one-year descriptive follow-up of bevacizumab treatment in hereditary haemorrhagic telangiectasia post-BABH interventional study. Ther Adv Hematol.

[30] Wang D, Ito S, Waldron C (2024). Cost-effectiveness of bevacizumab therapy in the care of patients with hereditary hemorrhagic telangiectasia. Blood Adv.

